# Multimodal Images of Acute Central Retinal Artery Occlusion

**DOI:** 10.1155/2017/5151972

**Published:** 2017-11-16

**Authors:** Parth Shah, Stephen G. Schwartz, Harry W. Flynn

**Affiliations:** Department of Ophthalmology, Bascom Palmer Eye Institute, University of Miami Miller School of Medicine, Miami, FL, USA

## Abstract

Two illustrative cases of acute central retinal artery occlusion (CRAO) are presented with multimodal imaging, including fluorescein angiography (FA) and commercially available optical coherence tomography angiography (OCT-A). In both patients, retinal ischemia was imaged well using both FA and OCT-A, and the two imaging studies provided comparable pictures. OCT-A provides useful information for the diagnosis and management of patients with acute CRAO, without the need for dye injection.

## 1. Introduction

Central retinal artery occlusion (CRAO) results from obstruction of blood flow due to embolic, thrombotic, inflammatory, or traumatic causes. In some eyes with CRAO, visual loss is relatively less severe due to sparing of the cilioretinal artery [1].

In most patients, the diagnosis of CRAO may be made with ophthalmoscopy alone, although ancillary testing is frequently used to confirm the diagnosis and to document the findings at presentation. Multimodal imaging includes fluorescein angiography (FA), spectral domain optical coherence tomography (SD-OCT), and optical coherence tomography angiography (OCT-A). Although FA has been used traditionally to evaluate the retinal circulation, OCT-A is an emerging technology that provides clinically useful information.

The present manuscript uses OCT-A to identify the pathologic features in two illustrative cases of CRAO. In both patients, OCT-A was performed using the commercially available Cirrus 5000 with AngioPlex (Zeiss, Jena, Germany) with no subsequent image processing. A 6 × 6 mm slab was used for all images.

## 2. Cases

### 2.1. Case 1

**Figure 1:**
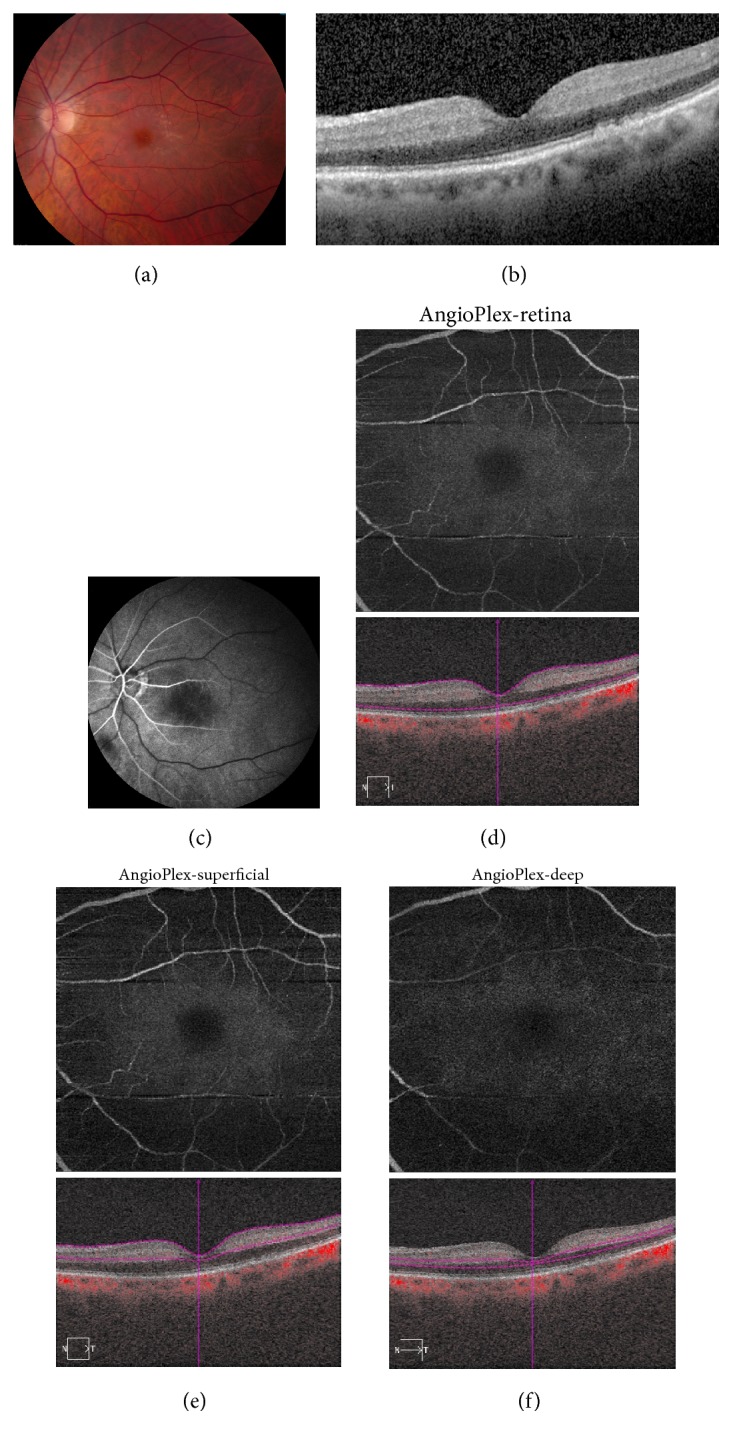
Acute central retinal artery occlusion, left eye. (a) Fundus photography reveals macular drusen and mild macular whitening with an early cherry red spot. (b) Spectral domain optical coherence tomography (SD-OCT) reveals thickening and hyperreflectivity of the inner retinal layers. (c) Fluorescein angiography (FA) at 26.58 seconds reveals delayed retinal perfusion. (d) Optical coherence tomography angiography (OCT-A) 6 × 6 mm retina slab reveals absent flow similar to that seen on FA. (e) OCT-A 6 × 6 mm superficial slab reveals absent flow. (f) OCT-A 6 × 6 mm deep slab reveals absent flow.

A 77-year-old male with a history of atrial fibrillation and nonneovascular age-related macular degeneration (AMD) presented about 4 hours following acute visual loss in the left eye. Best corrected visual acuity (BCVA) was count fingers. Fundus examination revealed macular drusen as well as mild macular whitening and an early cherry red spot (Figure 1(a)). SD-OCT demonstrated thickening and hyperreflectivity of the inner retinal layers (Figure 1(b)). FA at 26.58 seconds revealed delayed retinal perfusion (Figure 1(c)). The OCT-A retina slab (Figure 1(d)), superficial slab (Figure 1(e)), and deep slab (Figure 1(f)) revealed absent flow very similar to the FA.

### 2.2. Case 2

**Figure 2:**
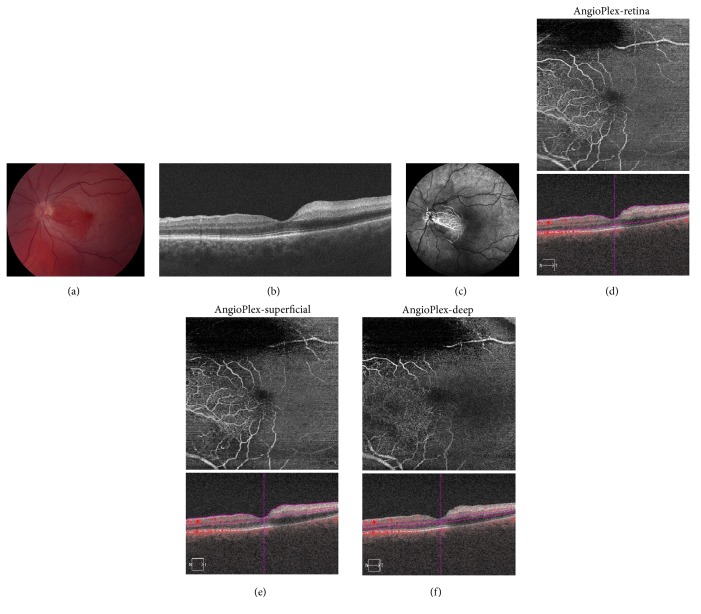
Acute central retinal artery occlusion with cilioretinal sparing, left eye. (a) Fundus photography reveals macular whitening with cilioretinal artery sparing. (b) Spectral domain optical coherence tomography (SD-OCT) reveals macular thickening and hyperreflectivity of the inner retinal layers temporal to the center of the macula, consistent with cilioretinal artery sparing. (c) Fluorescein angiography (FA) at 18.67 seconds reveals delayed retinal perfusion with cilioretinal artery sparing. (d) Optical coherence tomography angiography (OCT-A) 6 × 6 mm retina slab reveals absent flow similar to that seen on FA. There is diminished signal superiorly due to artifact. (e) OCT-A 6 × 6 mm superficial slab reveals absent flow. There is diminished signal superiorly due to artifact. (f) OCT-A 6 × 6 mm deep slab reveals absent flow. There is diminished signal superiorly due to artifact.

An 81-year-old male with a history of hypertension presented about 13 hours following acute visual loss in the left eye. BCVA was 20/50. Fundus examination revealed macular whitening in a pattern consistent with CRAO with cilioretinal artery sparing (Figure 2(a)). SD-OCT demonstrated thickening and hyperreflectivity of the inner retinal layers temporal to the center of the macula (Figure 2(b)). FA at 18.67 seconds revealed delayed retinal perfusion consistent with the pattern of macular whitening (Figure 2(c)). The OCT-A retina slab (6 × 6 mm) revealed absent flow in the same distribution as the FA (Figure 2(d)). There was diminished signal superiorly on the OCT-A due to artifact.

## 3. Discussion

OCT-A characteristics of CRAO have been reported previously [2–5]. These two cases illustrate the benefits of OCT-A in providing clinically useful information in the management of patients with acute CRAO without the need for fluorescein injection. In both patients, there is substantial concordance between the findings of the FA and the OCT-A performed on the same day. A similar concordance has been reported between FA and OCT-A for patients with chronic branch retinal vein occlusion [6].

OCT-A offers several advantages compared with traditional FA. OCT-A is noninvasive and has no risks of allergy [7]. In most patients, OCT-A can be obtained faster than FA. However, OCT-A is expensive and the image quality is affected by the patient's ability to fixate. In patients with poor vision, such as those with acute CRAO, it may not be possible to obtain good OCT-A images.

In patients with acute CRAO in whom an adequate OCT-A can be obtained, FA may not be necessary. Since many of these patients have serious systemic vascular diseases, OCT-A is an easily performed, quick, noninvasive alternative to FA.
